# Effects of Ephedrine-Containing Products on Weight Loss and Lipid Profiles: A Systematic Review and Meta-Analysis of Randomized Controlled Trials

**DOI:** 10.3390/ph14111198

**Published:** 2021-11-22

**Authors:** Hee-Jeong Yoo, Ha-Young Yoon, Jeong Yee, Hye-Sun Gwak

**Affiliations:** 1 College of Pharmacy and Graduate School of Pharmaceutical Sciences, Ewha Womans University, Seoul 03760, Korea; heejyoo@hanmail.net (H.-J.Y.); hayoungdymphnayoon@gmail.com (H.-Y.Y.); 2 Department of Pharmacy, National Medical Center, Seoul 04564, Korea

**Keywords:** mahuang, ephedrine, weight loss, blood pressure, lipid profiles, systematic review, meta-analysis

## Abstract

Ephedrine, the main active ingredient of mahuang, may lead to weight loss; however, it can also induce cardiovascular side effects. As ephedrine use remains controversial, this study aimed to systematically review previous studies on ephedrine-containing products and perform meta-analysis of the existing evidence on weight, blood pressure (BP), heart rate, and lipid change effects of ephedrine-containing products. We searched for placebo-controlled randomized studies in PubMed, Web of Science, and EMBASE until July 2021 using the following search terms: (ephedr* OR mahuang) AND (“weight loss” OR obes* OR overweight). Mean differences (MDs) and 95% confidence intervals (CIs) were calculated to evaluate the effects of ephedrine-containing products on weight, BP, heart rate, and lipid profiles. A total of 10 articles were included. Compared with the placebo group, the ephedrine-containing product group was associated with greater weight loss, with an MD of −1.97 kg (95% CI: −2.38, −1.57). In the ephedrine-containing product group, the mean heart rate was 5.76 beats/min higher than in the placebo group (95% CI: 3.42, 8.10), whereas intergroup differences in systolic and diastolic BP were not statistically significant. The ephedrine-containing product group had a significantly higher mean high-density lipoprotein cholesterol level (MD: 2.74 mg/dL; 95% CI: 0.94, 4.55), lower mean low-density lipoprotein cholesterol level (MD: −5.98 mg/dL; 95% CI: −10.97, −0.99), and lower mean triglyceride level (MD: −11.25 mg/dL; 95% CI: −21.83, −0.68) than the placebo group. Compared with placebo, the ephedrine-containing products showed better effects on weight loss and lipid profiles, whereas they caused increased heart rate. The ephedrine-containing products may be beneficial to obese or overweight patients; however, close monitoring is needed, especially heart rate monitoring.

## 1. Introduction

Obesity is a pathological condition in which excess fat in the body accumulates due to metabolic imbalance [1]. Obesity is recognized as one of the most important challenges facing public health and economies [2]. The prevalence of obesity has been continuously increasing. According to National Health and Nutrition Examination Survey (NHANES) data, over 40% of the American population is obese [1]. Additionally, obesity can lead to various diseases, such as diabetes mellitus, cardiovascular or musculoskeletal diseases, cancers, and psychiatric disorders [3,4,5,6].

Lifestyle modification, including calorie restriction and physical exercise, is the primary approach to weight loss [7]. Along with lifestyle changes, pharmacotherapy can be used to manage obesity, especially when body mass index (BMI) exceeds 30 kg/m^2^ or in the context of comorbid diseases and a BMI exceeding 27 kg/m^2^ [8]. Several drugs have been approved for obesity treatment, with sufficient evidence of safety and efficacy, including orlistat, phentermine–topiramate, naltrexone–bupropion and liraglutide [9]. Various herbal medicines and extracts (e.g., chitosan, garcinia, green tea, guar gum, mahuang, psyllium, and pyruvate) are also available for weight loss; however, unlike approved drugs, the clinical evidence for these products is not clear [10].

Mahuang, *Ephedra sinica*, is one of the most widely used herbal medicines for the treatment of obesity. Ephedrine promotes sympathetic neuronal actions, causing the heart to beat more strongly and quickly [11]. It also increases metabolism and suppresses appetite, thus promoting body fat decomposition [12,13]. Several studies have shown that single or mixed preparations of ephedrine can lead to weight loss and increased energy [14,15,16,17]. According to Douglas et al., patients taking ephedrine-containing capsules had a greater weight loss than those taking placebo (−3.1 kg vs. −2.1 kg in 8 weeks) [15]. Hackman et al. also showed that the ephedrine-treated groups lost more body weight than control groups (−3.0 kg vs. −1.0 kg in 3 months and −7.2 kg vs. −2.3 kg in 9 months) [17]. However, excessive ephedrine doses may cause adverse reactions, such as increased blood pressure and pulse rate, palpitations, insomnia, headaches, and nausea [18,19,20].

Several systematic reviews and meta-analyses have evaluated the effectiveness of herbal medicines for weight loss [21,22,23]. However, most studies have assessed the short-term efficacy of herbal medicines or have focused only on body weight and fat mass changes. Therefore, this systematic review and meta-analysis of studies investigating ephedrine-containing products aimed to summarize existing evidence regarding the effects of ephedrine-containing products on weight, in addition to new outcomes including blood pressure, heart rate, and lipid profiles.

## 2. Results

### 2.1. Literature Search

**Figure 1:**
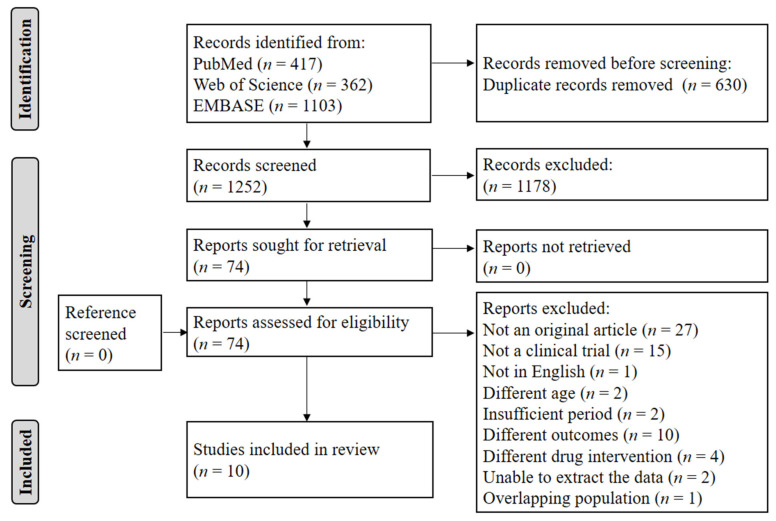
Article selection flow chart.

A detailed flow chart of the article selection process is presented in Figure 1. A total of 1882 articles were identified from searches of the three databases. After the removal of 630 duplicates, 1252 records were initially identified, of which the titles and abstracts were screened for inclusion. From this initial review, 74 articles were selected for full-text reviews and assessed for eligibility. Of these articles, 64 were excluded for the following reasons: not original research (*n* = 27), not a clinical trial (*n* = 15), not in English (*n* = 1), participants ineligible by age (*n* = 2), study duration less than 4 weeks (*n* = 2), ineligible outcomes (*n* = 10), ineligible drug interventions (*n* = 4), not amenable to data extraction (*n* = 2), and overlapping population (*n* = 1). Ultimately, 10 articles were selected for this systematic review [24,25,26,27,28,29,30,31,32,33].

The characteristics of the included articles are presented in Table 1. The articles were published between 1985 and 2015. Six of the studies were conducted in America, three in Europe, and one in Oceania. Daily ephedrine doses ranged from 60 to 150 mg and caffeine was co-administered in most studies. Study duration ranged between 4 and 24 weeks. The risk of bias was rated as “some concern” for two articles and “low” for eight articles.

### 2.2. Weight Loss

**Figure 2:**
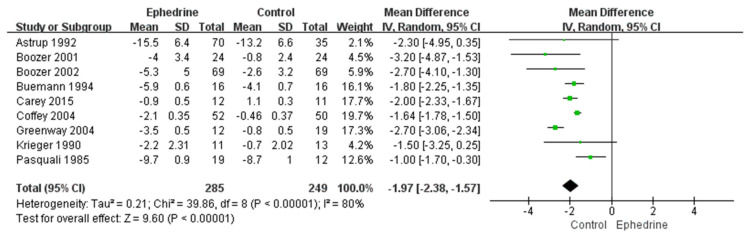
Forest plot to compare weight loss (kg) between ephedrine-containing products and placebo in obese or overweight patients.

Nine studies with 534 patients were evaluated to investigate the weight loss effect of ephedrine-containing products in obese or overweight individuals. The weight mean difference (MD) between the ephedrine-containing product group and the placebo group was −1.97 kg (95% confidence interval (CI): −2.38, −1.57; *p* < 0.00001; I^2^ = 80%) (Figure 2). Neither Begg’s test nor Egger’s test revealed significant publication bias (Begg’s test: *p* = 0.677; Egger’s test: *p* = 0.356; Figure S1, Supplementary Materials).

### 2.3. Blood Pressure and Heart Rate

**Figure 3:**
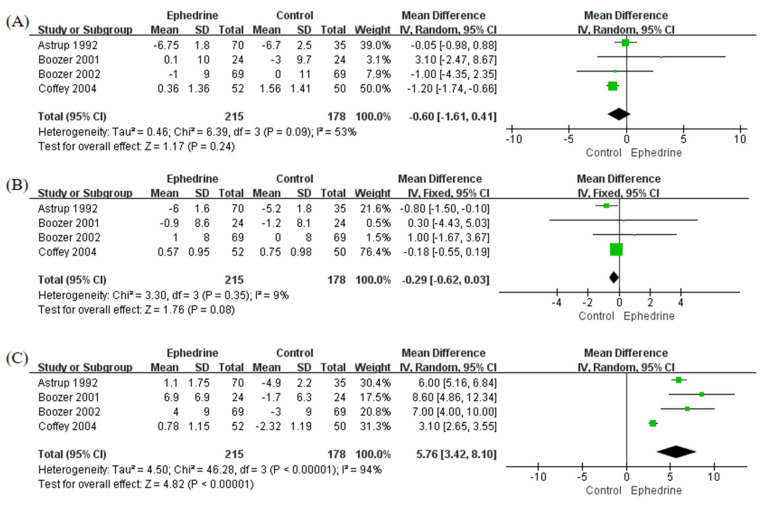
Forest plots to compare blood pressure and heart rate changes between ephedrine-containing products and placebo in obese or overweight patients. (**A**) Systolic blood pressure (mmHg); (**B**) diastolic blood pressure (mmHg); (**C**) heart rate (beats/min).

Four studies assessed the effects of ephedrine-containing products on blood pressure and heart rate. There were no significant differences in systolic blood pressure (SBP) and diastolic blood pressure (DBP) between the ephedrine-based treatment group and the placebo group (Figure 3A,B, respectively). In contrast, the mean heart rate of the ephedrine-containing product group was 5.76 beats/min higher than that of the placebo group (95% CI: 3.42, 8.10; *p* < 0.00001; I^2^ = 94%) (Figure 3C). No significant publication biases were detected in any blood pressure or heart rate analyses, and all *p* values of the Begg’s and Egger’s tests were >0.05 (Figure S2, Supplementary Materials).

### 2.4. Lipid Levels

**Figure 4:**
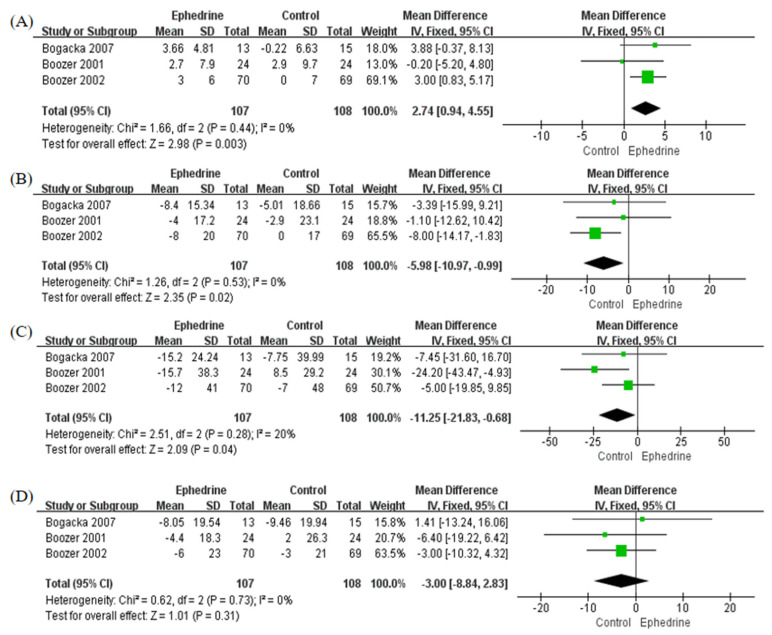
Forest plots to compare lipid profile changes between ephedrine-containing products and placebo in obese or overweight patients. (**A**) High-density lipoprotein cholesterol (mg/dL); (**B**) low-density lipoprotein cholesterol (mg/dL); (**C**) triglycerides (mg/dL); (**D**) total cholesterol (mg/dL).

Three studies investigated the influence of ephedrine-containing products on high-density lipoprotein cholesterol (HDL-C), low-density lipoprotein cholesterol (LDL-C), triglyceride (TG), or total cholesterol (TC). The ephedrine-containing products group had a higher mean HDL-C concentration, with an MD of 2.74 mg/dL (95% CI: 0.94, 4.55; *p* = 0.003; I^2^ = 0%) (Figure 4A). Additionally, the ephedrine-containing product group had significantly lower mean concentrations of LDL-C (Figure 4B) and TG (Figure 4C) than the placebo group: the MDs were −5.98 mg/dL (95% CI: −10.97, −0.99; *p* = 0.02; I^2^ = 0%) and −11.25 mg/dL (95% CI: −21.83, −0.68; *p* = 0.04; I^2^ = 20%), respectively. In terms of TC, there was no significant intergroup difference, but the ephedrine-containing product group had a lower mean TC concentration (MD: −3.00; 95% CI: −8.84, 2.83; *p* = 0.31; I^2^ = 0%, Figure 4D). Neither Begg’s test nor Egger’s test showed significant publication bias in any of the lipid level analyses (Figure S3, Supplementary Materials).

### 2.5. Subgroup Analysis

Among the included studies, six studies were published after the year 2000; there were five studies for weight loss and three studies for change of blood pressure and heart rate. In terms of lipid levels, all studies were published after the year 2000. The weight MD between the ephedrine-containing product group and the placebo group was −2.25 kg (95% CI: −2.81, −1.69; *p* < 0.00001; I^2^ = 88%; Figure S4, Supplementary Materials). The SBP of the ephedrine-containing product group was 1.16 mmHg lower than that of the placebo group (95% CI: −1.69, −0.63; *p* < 0.0001; I^2^ = 12%), whereas there was no significant difference in DBP between the ephedrine-based treatment group and the placebo group. In terms of heart rate, the ephedrine-containing product group had 5.90 beats/min higher heart rate than that of the placebo group (95% CI: 2.18, 9.63; *p* = 0.0008; I^2^ = 86%).

## 3. Discussion

In this systematic review and meta-analysis, we demonstrated the effects of ephedrine-containing products on weight loss, BP, heart rate, and lipid profiles in obese or overweight patients. The main results are as follows: compared with placebo, the ephedrine-containing products in daily dose from 60 to 150 mg for 4 to 24 weeks (1) significantly decreased the body weight with an MD of −2.0 kg, (2) increased the heart rate by 5.8 beats/min, and (3) were associated with better lipid profiles (decreased LDL-C, TG, and TC with an MD of −6.0 mg/dL, −11.3 mg/dL, and −3.0 mg/dL, respectively, and increased HDL-C by 2.7 mg/dL). The subgroup analysis with studies published after the year 2000 showed a similar trend as the main results. As the subgroup analysis included only three to five studies, it should be further validated.

Several meta-analyses have investigated the weight loss effects of prescription drugs and complementary medicines for weight management. A recent network meta-analysis showed that, compared with placebo, orlistat and lorcaserin were associated with mean weight loss of around 3 kg over 1 year, and naltrexone–bupropion and phentermine–topiramate were associated with mean weight loss of 5 kg and 9 kg in a year, respectively [34]. Another meta-analysis investigated the weight loss effects of complementary medicines and found that green tea-containing and garcinia-containing herbal medicines achieved 1.6 kg and 0.4 kg of weight loss, respectively. However, single preparations of each herbal extract were not associated with statistically significant changes [22]. Our study revealed that ephedrine-containing products were associated with around 2 kg of weight loss, which was greater than the extent of weight reduction associated with other herbal medicines but lower than that associated with weight loss drugs. Although a clinically significant weight loss is usually regarded as 2.5 kg or more, which is known to be sufficient for reducing cardiovascular events and mortality in large trials [35], the weight loss effect of ephedrine-containing products was quite beneficial for obesity management.

Ephedrine, a mixed sympathomimetic agent, stimulates α- and β-adrenergic receptors and increases the release of norepinephrine [36]. It has been known to increase myocardial contractility and heart rate and raise BP [37]. Due to its adverse cardiovascular effects, the U.S. Food and Drug Administration (FDA) prohibited the sale of dietary supplements containing ephedrine alkaloids in 2004 [38]. However, 1 year later, the U.S. District Court overturned the FDA’s ban [39], and the cardiovascular safety of ephedrine remained to be clarified. Our meta-analysis results show that patients taking ephedrine-containing products had faster heart rates, which was consistent with previous studies showing that ephedrine-containing dietary supplements were associated with a 2-fold higher risk of palpitations [20]. However, our review failed to demonstrate statistical significance in terms of ephedrine’s effects on SBP and DBP, possibly due to the small number of included studies. Further research is warranted in this regard.

Ephedrine-containing products were also shown to be associated with improved lipid profiles. This finding was similar to that reported after an in vivo study in which the ephedra group had lower TG and higher HDL levels [40]. This can be explained by the differential expression of lipid metabolism-related genes. Matteis et al. report that ephedrine with caffeine increases the expression of the β3-adrenergic receptor in adipocytes, which are involved in lipolysis [41]. It has also been shown that mice treated with ephedra extracts have elevated expression of PPAR-α and adiponectin, which are known to regulate lipid and lipoprotein metabolism [40]. Accordingly, ephedrine is likely to decrease fat accumulation, thereby leading to weight loss.

This review had several limitations. First, the daily dose of ephedrine varied from study to study, leading to clinical heterogeneity. Second, most studies co-administered caffeine, which is also known to be beneficial for weight control by increasing energy expenditure and decreasing energy intake [42,43]. There may be synergistic effects on weight loss, which makes it difficult to distinguish their own effects on weight loss. Third, some potential confounders, such as age, baseline body weight, and comorbidities, could not be adjusted for in the analysis. Fourth, as we did not limit the publication year of studies, some studies were quite old, with around half of them performed before the year 2000. Last, this meta-analysis only included articles reporting on studies of obese or overweight adults; therefore, the results should be interpreted with caution, especially for adolescents, older adults, and non-obese populations.

## 4. Materials and Methods

### 4.1. Literature Search Strategy and Inclusion Criteria

This meta-analysis was conducted according to the checklist outlined in the Preferred Reporting Items for Systematic Reviews and Meta-Analyses (PRISMA) [44]. An extensive search was performed using three electronic databases (PubMed, Web of Science, and EMBASE) until July 2021 using the following search terms: (ephedr* OR mahuang) AND (“weight loss” OR obes* OR overweight). 

Articles were eligible for inclusion if they (i) reported on original research and were published in peer-reviewed journals; (ii) involved obese or overweight adults (>18 years of age); (iii) compared ephedrine-containing products with placebo; (iv) evaluated one or more clinical outcomes, including weight, heart rate, blood pressure (SBP or DBP), or lipid levels (HDL-C, LDL-C, TG, or TC); (v) followed participants for at least 4 weeks; and (vi) were randomized controlled trials, including parallel or crossover designs. Articles were excluded if they (i) were in vitro or animal studies, (ii) were not published in English, or (iii) were not amenable to data extraction. If multiple articles had overlapping data, only the one article that included the most comprehensive data was included in the meta-analysis.

### 4.2. Data Extraction and Study Quality Assessment

Two reviewers independently extracted data, and discrepancies were resolved by discussions until consensus was reached. The following information was extracted: first author’s name, publication year, country, number of patients, percentage of males, mean age and baseline body weight, intervention, and study duration.

Two researchers independently assessed the selected articles according to the revised Cochrane risk-of-bias tool for randomized trials (RoB 2.0) [45] with the following five domains: (1) randomization process, (2) deviations from intended interventions, (3) missing outcome data, (4) measurement of the outcome, and (5) selection of the reported result. Articles were rated as “high”, “low”, or “some concerns” in terms of each of these domains. Any disagreement was resolved through discussion between the two assessors or, when necessary, in consultation with a third investigator.

### 4.3. Statistical Analysis

MDs and 95% CIs were calculated to identify the effects of ephedrine-containing products on weight, blood pressure, heart rate, and lipid levels. Heterogeneity across studies was estimated by way of chi-square and I² analyses. An I² value ≥50% indicated significant heterogeneity. A fixed-effects model was used if I² was <50%, and a random-effects model was used if I² was ≥50% [46]. Both Begg’s rank correlation test and Egger’s regression test of the funnel plot were conducted to detect publication bias [47,48]. A subgroup analysis was performed with studies published after the year 2000. Statistical analyses were performed using Review Manager (version 5.3; The Cochrane Collaboration, Copenhagen, Denmark) and R Studio software (version 3.6.0; R Foundation for Statistical Computing, Vienna, Austria). A *p*-value <0.05 was considered statistically significant.

## 5. Conclusions

This systematic review and meta-analysis provides sufficient evidence concerning the weight loss and lipid-modifying effects of ephedrine-containing products, along with the possibility of increasing heart rate. Ephedrine-containing products may benefit obese or overweight individuals; however, close monitoring is crucial, particularly heart rate monitoring.

## Figures and Tables

**Table 1:** Summary of included articles.

Study	Country	Population (Male %)	Age (year)	Baseline Body Weight (kg)	Experimental Group	Duration	Risk of Bias
Astrup 1992	Denmark	135 (16.3)	28.3 ± 7.0	95.1 ± 14.4	Ephedrine 20 mg and caffeine 200 mg tid, ephedrine 20 mg tid	24 weeks	Low
Bogacka 2007	USA	41 (17.1)	37.5 ± 7.8	94.1 ± 11.4	Ephedrine 25 mg and caffeine 200 mg tid	16 weeks	Some concerns
Boozer 2001	USA	67 (14.9)	41.1 ± 8.7	89.4 ± 10.3	Ephedrine 24 mg and caffeine 80 mg tid	8 weeks	Low
Boozer 2002	USA	167 (18.0)	45.3 ± 12.3	88.0 ± 14.3	Ephedrine 30 mg and caffeine 64 mg tid	6 months	Low
Buemann 1994	Denmark	32 (0.0)	N/A	93.3 ± 2.9	Ephedrine 20 mg and caffeine 200 mg tid	8 weeks	Low
Carey 2015	Australia	23 (100.0)	22.5 ± 1.6	77.1 ± 2.6	Day 1: ephedrine 2.5 mg/kg, day 2–28: ephedrine 1.5 mg/kg/day	28 days	Some concerns
Coffey 2004	USA	102 (13.7)	43.5 ± 10.0	92.8 ± 12.0	Ephedrine 10 mg, caffeine 60 mg, and salicin 15 mg (N/A)	12 weeks	Low
Greenway 2004	USA	40 (17.5)	46.1 ± 2.4	83.0 ± 2.7	Ephedrine 24 mg and caffeine 60 mg tid	3 months	Low
Krieger 1990	USA	24 (8.3)	35.4 ± 7.5	N/A	Week 1–4: ephedrine 25 mg, caffeine 50 mg, and aspirin 100 mg tid, week 5–8: ephedrine 50 mg, caffeine 50 mg, and aspirin 100 mg tid	8 weeks	Low
Pasquali 1985	Italy	46 (30.4)	36.3 ± 3.8	N/A	Ephedrine 25 mg tid, ephedrine 50 mg tid	3 months	Low

N/A: not available, qd: once a day, tid: three times a day, USA: United States of America.

## Data Availability

Data sharing is not applicable to this article.

## Supplementary Materials

The following are available online at https://www.mdpi.com/article/10.3390/ph14111198/s1, Figure S1: Funnel plot demonstrating the association between ephedrine-containing products and weight loss (kg), Figure S2: Funnel plots demonstrating the association between ephedrine-containing products and vital sign change, Figure S3: Funnel plots demonstrating the association between ephedrine-containing products and lipid level change, Figure S4: Forest plots of subgroup analysis with studies published after the year 2000: (A) weight loss (kg); (B) systolic blood pressure (mmHg); (C) diastolic blood pressure (mmHg); (D) heart rate (beats/min).

## References

[1] Ogden C.L., Fryar C.D., Martin C.B., Freedman D.S., Carroll M.D., Gu Q., Hales C.M. (2020). Trends in Obesity Prevalence by Race and Hispanic Origin-1999–2000 to 2017–2018. JAMA.

[2] Noncommunicable Diseases. https://www.who.int/en/news-room/fact-sheets/detail/noncommunicable-diseases.

[3] Freemantle N., Holmes J., Hockey A., Kumar S. (2008). How strong is the association between abdominal obesity and the incidence of type 2 diabetes?. Int. J. Clin. Pract..

[4] Bhaskaran K., Douglas I., Forbes H., dos-Santos-Silva I., Leon D.A., Smeeth L. (2014). Body-mass index and risk of 22 specific cancers: A population-based cohort study of 5·24 million UK adults. Lancet.

[5] Ni Mhurchu C., Rodgers A., Pan W.H., Gu D.F., Woodward M., Asia Pacific Cohort Studies Collaboration (2004). Body mass index and cardiovascular disease in the Asia-Pacific Region: An overview of 33 cohorts involving 310,000 participants. Int. J. Epidemiol..

[6] Kopelman P. (2007). Health risks associated with overweight and obesity. Obes. Rev..

[7] Wadden T.A., Butryn M.L., Byrne K.J. (2004). Efficacy of lifestyle modification for long-term weight control. Obes. Res..

[8] Apovian C.M., Aronne L.J., Bessesen D.H., McDonnell M.E., Murad M.H., Pagotto U., Ryan D., Still C.D. (2015). Pharmacological management of obesity: An endocrine Society clinical practice guideline. J. Clin. Endocrinol. Metab..

[9] Daneschvar H.L., Aronson M.D., Smetana G.W. (2016). FDA-Approved Anti-Obesity Drugs in the United States. Am. J. Med..

[10] Saper R.B., Eisenberg D.M., Phillips R.S. (2004). Common dietary supplements for weight loss. Am. Fam. Physician.

[11] Persky A.M., Berry N.S., Pollack G.M., Brouwer K.L. (2004). Modelling the cardiovascular effects of ephedrine. Br. J. Clin. Pharmacol..

[12] Diepvens K., Westerterp K.R., Westerterp-Plantenga M.S. (2007). Obesity and thermogenesis related to the consumption of caffeine, ephedrine, capsaicin, and green tea. Am. J. Physiol. Regul. Integr. Comp. Physiol..

[13] Vallerand A.L., Jacobs I., Kavanagh M.F. (1989). Mechanism of enhanced cold tolerance by an ephedrine-caffeine mixture in humans. J. Appl. Physiol..

[14] Ingerslev J., Svendsen T.L., Mørk A. (1997). Is an ephedrine caffeine treatment contraindicated in hypertension?. Int. J. Obes. Relat. Metab. Disord..

[15] Kalman D.S., Colker C.M., Shi Q., Swain M.A. (2000). Effects of a weight-loss aid in healthy overweight adults: Double-blind. Curr. Ther. Res..

[16] Hioki C., Yoshimoto K., Yoshida T. (2004). Efficacy of bofu-tsusho-san, an oriental herbal medicine, in obese Japanese women with impaired glucose tolerance. Clin. Exp. Pharmacol. Physiol..

[17] Hackman R.M., Havel P.J., Schwartz H.J., Rutledge J.C., Watnik M.R., Noceti E.M., Stohs S.J., Stern J.S., Keen C.L. (2006). Multinutrient supplement containing ephedra and caffeine causes weight loss and improves metabolic risk factors in obese women: A randomized controlled trial. Int. J. Obes. (Lond.).

[18] Laccourreye O., Werner A., Giroud J.P., Couloigner V., Bonfils P., Bondon-Guitton E. (2015). Benefits, limits and danger of ephedrine and pseudoephedrine as nasal decongestants. Eur. Ann. Otorhinolaryngol. Head Neck Dis..

[19] Wooltorton E., Sibbald B. (2002). Ephedra/ephedrine: Cardiovascular and CNS effects. CMAJ.

[20] Shekelle P.G., Hardy M.L., Morton S.C., Maglione M., Mojica W.A., Suttorp M.J., Rhodes S.L., Jungvig L., Gagné J. (2003). Efficacy and safety of ephedra and ephedrine for weight loss and athletic performance: A meta-analysis. JAMA.

[21] Hasani-Ranjbar S., Nayebi N., Larijani B., Abdollahi M. (2009). A systematic review of the efficacy and safety of herbal medicines used in the treatment of obesity. World J. Gastroenterol..

[22] Maunder A., Bessell E., Lauche R., Adams J., Sainsbury A., Fuller N.R. (2020). Effectiveness of herbal medicines for weight loss: A systematic review and meta-analysis of randomized controlled trials. Diabetes Obes. Metab..

[23] Batsis J.A., Apolzan J.W., Bagley P.J., Blunt H.B., Divan V., Gill S., Golden A., Gundumraj S., Heymsfield S.B., Kahan S. (2021). A Systematic Review of Dietary Supplements and Alternative Therapies for Weight Loss. Obes. (Silver Spring).

[24] Astrup A., Breum L., Toubro S., Hein P., Quaade F. (1992). The effect and safety of an ephedrine/caffeine compound compared to ephedrine, caffeine and placebo in obese subjects on an energy restricted diet. A double blind trial. Int. J. Obes. Relat. Metab. Disord..

[25] Bogacka I., Gettys T.W., de Jonge L., Nguyen T., Smith J.M., Xie H., Greenway F., Smith S.R. (2007). The effect of beta-adrenergic and peroxisome proliferator-activated receptor-gamma stimulation on target genes related to lipid metabolism in human subcutaneous adipose tissue. Diabetes Care.

[26] Boozer C.N., Nasser J.A., Heymsfield S.B., Wang V., Chen G., Solomon J.L. (2001). An herbal supplement containing Ma Huang-Guarana for weight loss: A randomized, double-blind trial. Int. J. Obes. Relat. Metab. Disord..

[27] Boozer C.N., Daly P.A., Homel P., Solomon J.L., Blanchard D., Nasser J.A., Strauss R., Meredith T. (2002). Herbal ephedra/caffeine for weight loss: A 6-month randomized safety and efficacy trial. Int. J. Obes. Relat. Metab. Disord..

[28] Buemann B., Marckmann P., Christensen N.J., Astrup A. (1994). The effect of ephedrine plus caffeine on plasma lipids and lipoproteins during a 4.2 MJ/day diet. Int. J. Obes. Relat. Metab. Disord..

[29] Carey A.L., Pajtak R., Formosa M.F., Van Every B., Bertovic D.A., Anderson M.J., Eikelis N., Lambert G.W., Kalff V., Duffy S.J. (2015). Chronic ephedrine administration decreases brown adipose tissue activity in a randomised controlled human trial: Implications for obesity. Diabetologia.

[30] Coffey C.S., Steiner D., Baker B.A., Allison D.B. (2004). A randomized double-blind placebo-controlled clinical trial of a product containing ephedrine, caffeine, and other ingredients from herbal sources for treatment of overweight and obesity in the absence of lifestyle treatment. Int. J. Obes. Relat. Metab. Disord..

[31] Greenway F.L., De Jonge L., Blanchard D., Frisard M., Smith S.R. (2004). Effect of a dietary herbal supplement containing caffeine and ephedra on weight, metabolic rate, and body composition. Obes. Res..

[32] Krieger D.R., Daly P.A., Dulloo A.G., Ransil B.J., Young J.B., Landsberg L. (1990). Ephedrine, caffeine and aspirin promote weight loss in obese subjects. Trans. Assoc. Am. Physicians.

[33] Pasquali R., Baraldi G., Cesari M.P., Melchionda N., Zamboni M., Stefanini C., Raitano A. (1985). A controlled trial using ephedrine in the treatment of obesity. Int. J. Obes..

[34] Khera R., Murad M.H., Chandar A.K., Dulai P.S., Wang Z., Prokop L.J., Loomba R., Camilleri M., Singh S. (2016). Association of Pharmacological Treatments for Obesity With Weight Loss and Adverse Events: A Systematic Review and Meta-analysis. JAMA.

[35] Caterson I.D., Finer N., Coutinho W., Van Gaal L.F., Maggioni A.P., Torp-Pedersen C., Sharma A.M., Legler U.F., Shepherd G.M., Rode R.A. (2012). Maintained intentional weight loss reduces cardiovascular outcomes: Results from the Sibutramine Cardiovascular OUTcomes (SCOUT) trial. Diabetes Obes. Metab..

[36] Westfall T.C., Macarthur H., Westfall D.P., Brunton L.L., Hilal-Dandan R., Knollmann B.C. (2017). Adrenergic agonists and antagonists. Goodman & Gilman’s: The Pharmacological Basis of Therapeutics.

[37] Haller C.A., Benowitz N.L. (2000). Adverse cardiovascular and central nervous system events associated with dietary supplements containing ephedra alkaloids. N. Engl. J. Med..

[38] Food and Drug Administration HHS (2004). Final rule declaring dietary supplements containing ephedrine alkaloids adulterated because they present an unreasonable risk Final rule. Fed. Regist..

[39] Tynes J.R. (2006). Performance enhancing substances. Effects, regulations, and the pervasive efforts to control doping in Major League Baseball. J. Leg. Med..

[40] Song M.K., Um J.Y., Jang H.J., Lee B.C. (2012). Beneficial effect of dietary Ephedra sinica on obesity and glucose intolerance in high-fat diet-fed mice. Exp. Ther. Med..

[41] De Matteis R., Arch J.R., Petroni M.L., Ferrari D., Cinti S., Stock M.J. (2002). Immunohistochemical identification of the beta(3)-adrenoceptor in intact human adipocytes and ventricular myocardium: Effect of obesity and treatment with ephedrine and caffeine. Int. J. Obes. Relat. Metab. Disord..

[42] Harpaz E., Tamir S., Weinstein A., Weinstein Y. (2017). The effect of caffeine on energy balance. J. Basic Clin. Physiol. Pharmacol..

[43] Schubert M.M., Hall S., Leveritt M., Grant G., Sabapathy S., Desbrow B. (2014). Caffeine consumption around an exercise bout: Effects on energy expenditure, energy intake, and exercise enjoyment. J. Appl. Physiol..

[44] Page M.J., McKenzie J.E., Bossuyt P.M., Boutron I., Hoffmann T.C., Mulrow C.D., Shamseer L., Tetzlaff J.M., Akl E.A., Brennan S.E. (2021). The PRISMA 2020 statement: An updated guideline for reporting systematic reviews. BMJ.

[45] Sterne J.A.C., Savović J., Page M.J., Elbers R.G., Blencowe N.S., Boutron I., Cates C.J., Cheng H.-Y., Corbett M.S., Eldridge S.M. (2019). RoB 2: A revised tool for assessing risk of bias in randomised trials. BMJ.

[46] DerSimonian R., Laird N. (1986). Meta-analysis in clinical trials. Control Clin. Trials.

[47] Begg C.B., Mazumdar M. (1994). Operating characteristics of a rank correlation test for publication bias. Biometrics.

[48] Egger M., Davey Smith G., Schneider M., Minder C. (1997). Bias in meta-analysis detected by a simple, graphical test. BMJ.

