# Supplementary material for: Effects of Ephedrine-Containing Products on Weight Loss and Lipid Profiles: A Systematic Review and Meta-Analysis of Randomized Controlled Trials
Source: Pharmaceuticals (Basel). 2021 Nov 22;14(11):1198. doi: 10.3390/ph14111198 (PMC8618781; doi:10.3390/ph14111198)
Supplement: Supplementary file 1 [file pharmaceuticals-14-01198-s001.zip › pharmaceuticals-1429537-supplementary.pdf]

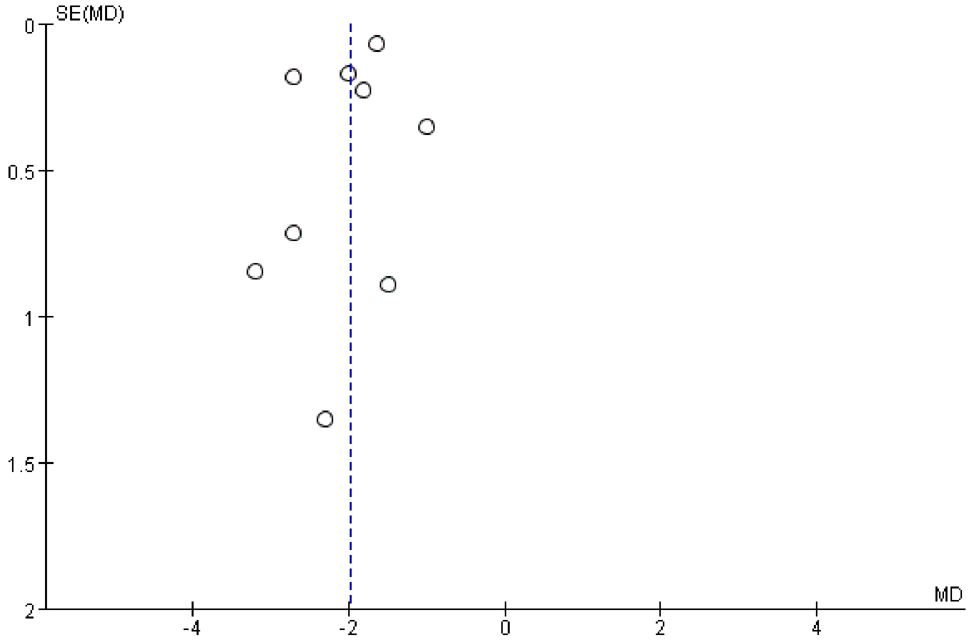

Figure S1. Funnel plot demonstrating the association between ephedrine-containing product and weight loss (kg).

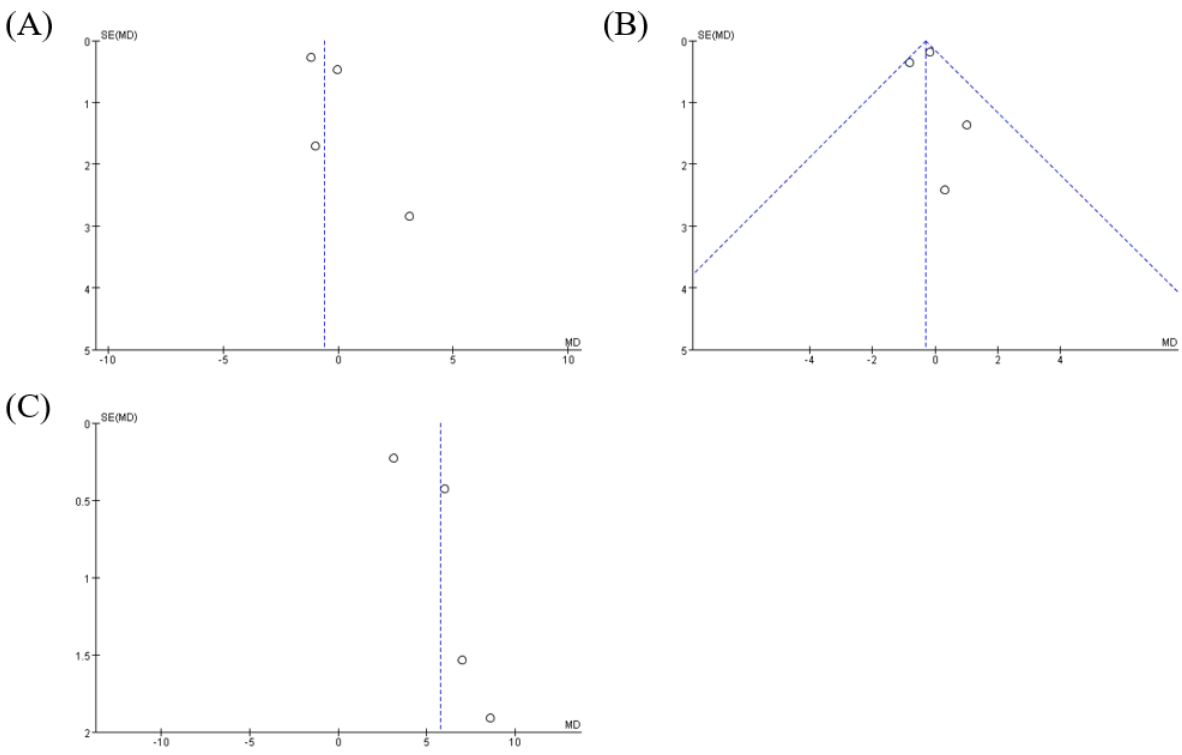

Figure S2. Funnel plots demonstrating the association between ephedrine-containing product and vital sign change. (A) Systolic blood pressure (mmHg). (B) Diastolic blood pressure (mmHg). (C) Heart rate (beats/min).

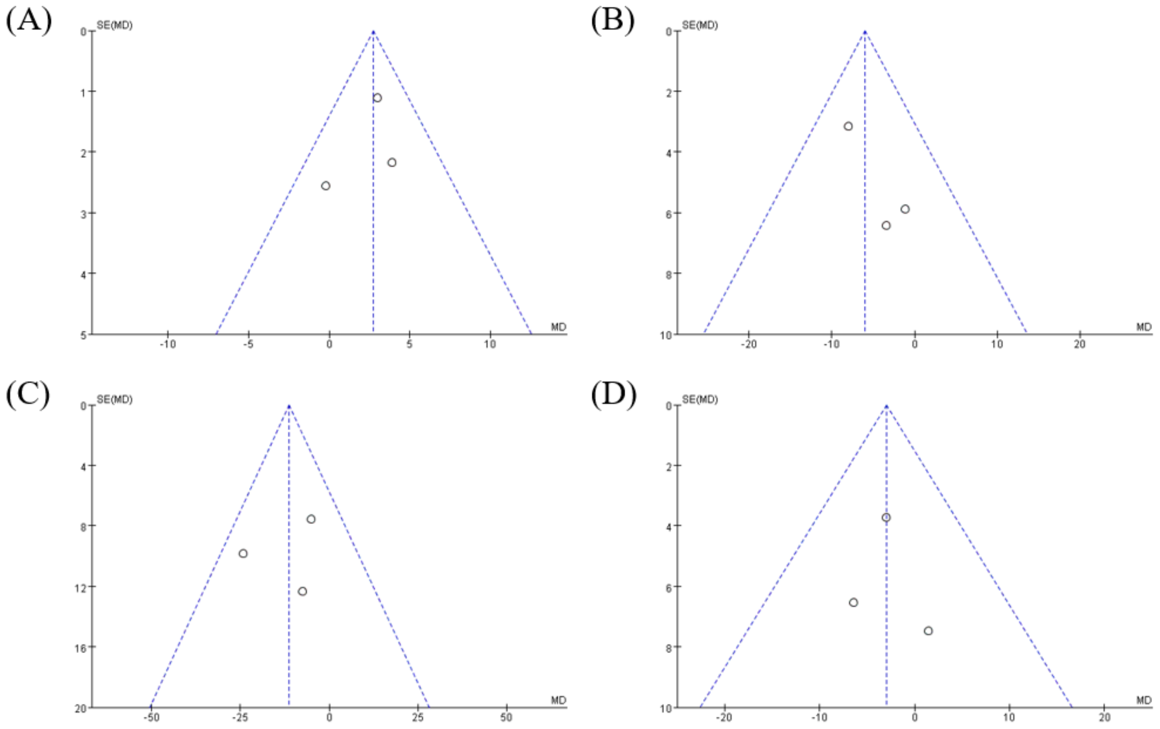

Figure S3. Funnel plots demonstrating the association between ephedrine-containing product and lipid level change. (A) High-density lipoprotein cholesterol (mg/dL). (B) Low-density lipoprotein cholesterol (mg/dL). (C) Triglycerides (mg/dL). (D) Total cholesterol (mg/dL).

(A)

Study or Subgroup	Ephedrine Mean	Ephedrine SD	Ephedrine Total	Control Mean	Control SD	Control Total	Weight	Mean Difference IV, Random, 95% CI
Boozer 2001	-4	3.4	24	-0.8	2.4	24	8.2%	-3.20 [-4.87, -1.53]
Boozer 2002	-5.3	5	69	-2.6	3.2	69	10.3%	-2.70 [-4.10, -1.30]
Carey 2015	-0.9	0.5	12	1.1	0.3	11	26.6%	-2.00 [-2.33, -1.67]
Coffey 2004	-2.1	0.35	52	-0.46	0.37	50	28.8%	-1.64 [-1.78, -1.50]
Greenway 2004	-3.5	0.5	12	-0.8	0.5	19	26.1%	-2.70 [-3.06, -2.34]
**Total (95% CI)**			**169**			**173**	**100.0%**	**-2.25 [-2.81, -1.69]**

Heterogeneity: Tau² = 0.28; Chi² = 34.41, df = 4 (P < 0.00001); I² = 88%

Test for overall effect: Z = 7.88 (P < 0.00001)

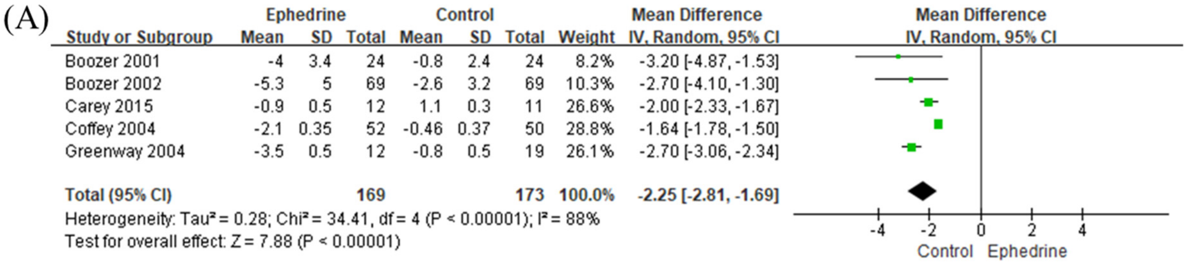

(B)

Study or Subgroup	Ephedrine Mean	Ephedrine SD	Ephedrine Total	Control Mean	Control SD	Control Total	Weight	Mean Difference IV, Fixed, 95% CI
Boozer 2001	0.1	10	24	-3	9.7	24	0.9%	3.10 [-2.47, 8.67]
Boozer 2002	-1	9	69	0	11	69	2.5%	-1.00 [-4.35, 2.35]
Coffey 2004	0.36	1.36	52	1.56	1.41	50	96.6%	-1.20 [-1.74, -0.66]
**Total (95% CI)**			**145**			**143**	**100.0%**	**-1.16 [-1.69, -0.63]**

Heterogeneity: Chi² = 2.27, df = 2 (P = 0.32); I² = 12%

Test for overall effect: Z = 4.29 (P < 0.0001)

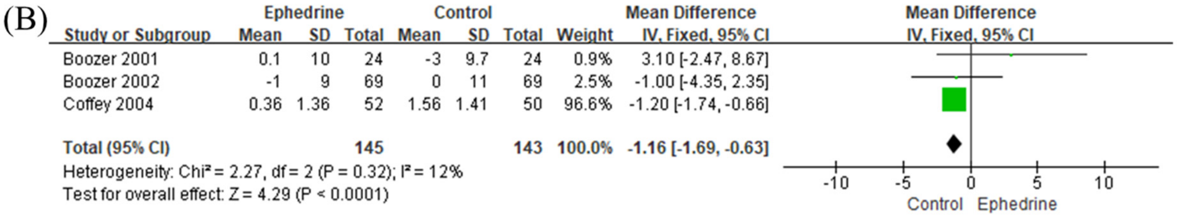

(C)

Study or Subgroup	Ephedrine Mean	Ephedrine SD	Ephedrine Total	Control Mean	Control SD	Control Total	Weight	Mean Difference IV, Random, 95% CI
Boozer 2001	-0.9	8.6	24	-1.2	8.1	24	0.6%	0.30 [-4.43, 5.03]
Boozer 2002	1	8	69	0	8	69	1.9%	1.00 [-1.67, 3.67]
Coffey 2004	0.57	0.95	52	0.75	0.98	50	97.5%	-0.18 [-0.55, 0.19]
**Total (95% CI)**			**145**			**143**	**100.0%**	**-0.15 [-0.52, 0.22]**

Heterogeneity: Tau² = 0.00; Chi² = 0.77, df = 2 (P = 0.68); I² = 0%

Test for overall effect: Z = 0.82 (P = 0.41)

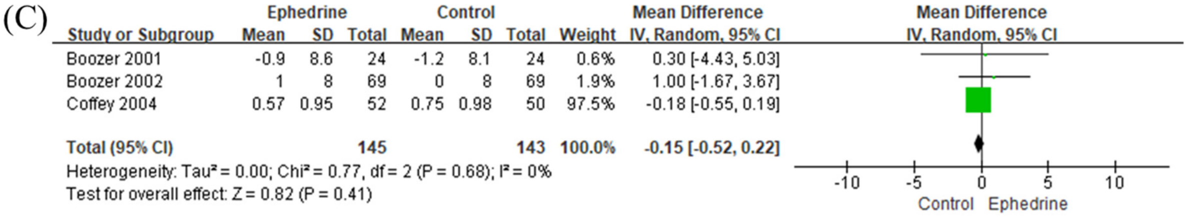

(D)

Study or Subgroup	Ephedrine Mean	Ephedrine SD	Ephedrine Total	Control Mean	Control SD	Control Total	Weight	Mean Difference IV, Random, 95% CI
Boozer 2001	6.9	6.9	24	-1.7	6.3	24	28.5%	8.60 [4.86, 12.34]
Boozer 2002	4	9	69	-3	9	69	31.7%	7.00 [4.00, 10.00]
Coffey 2004	0.78	1.15	52	-2.32	1.19	50	39.8%	3.10 [2.65, 3.55]
**Total (95% CI)**			**145**			**143**	**100.0%**	**5.90 [2.18, 9.63]**

Heterogeneity: Tau² = 9.00; Chi² = 14.27, df = 2 (P = 0.0008); I² = 86%

Test for overall effect: Z = 3.11 (P = 0.002)

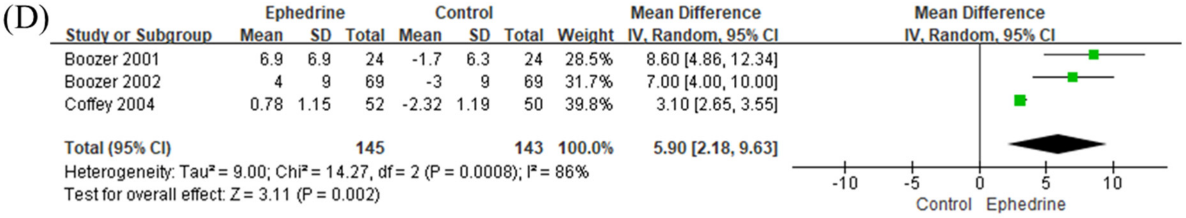

Figure S4. Forest plots of subgroup analysis with studies published after 2000s. (A) weight loss (kg); (B) systolic blood pressure (mmHg); (C) diastolic blood pressure (mmHg); (D) heart rate (beats/min).
